# X-ray diffraction from strongly bent crystals and spectroscopy of X-ray free-electron laser pulses

**DOI:** 10.1107/S2053273319014347

**Published:** 2020-01-01

**Authors:** Vladimir M. Kaganer, Ilia Petrov, Liubov Samoylova

**Affiliations:** a Paul-Drude-Institut für Festkörperelektronik, Leibniz-Institut im Forschungsverbund Berlin e.V., Hausvogteiplatz 5–7, 10117 Berlin, Germany; b European XFEL GmbH, Holzkoppel 4, 22869 Schenefeld, Germany

**Keywords:** X-ray free-electron lasers, X-ray spectroscopy, bent crystals, diamond crystal optics, femtosecond X-ray diffraction, dynamical diffraction

## Abstract

The use of strongly bent crystals in spectrometers for pulses of a hard X-ray free-electron laser is explored theoretically. Diffraction is calculated in both dynamical and kinematical theories. It is shown that diffraction can be treated kinematically when the bending radius is small compared with the critical radius given by the ratio of the Bragg-case extinction length for the actual reflection to the Darwin width of this reflection. As a result, the spectral resolution is limited by the crystal thickness, rather than the extinction length, and can become better than the resolution of a planar dynamically diffracting crystal. As an example, it is demonstrated that spectra of the 12 keV pulses can be resolved in the 440 reflection from a 20 µm-thick diamond crystal bent to a radius of 10 cm.

## Abstract 2

A strongly bent crystal diffracts kinematically when the bending radius is small compared with the critical radius given by the ratio of the extinction length to the Darwin width of the reflection. Under these conditions, the spectral resolution of the X-ray free-electron laser pulse is limited by the crystal thickness and can be better than under dynamical diffraction conditions.

## 1. Introduction   

Bent single crystals are commonly used as the X-ray optic elements for beam conditioning as well as the analysers for X-ray spectroscopy. The dynamical diffraction from bent crystals has been a topic of numerous studies over decades (Penning & Polder, 1961 ▸; Kato, 1964 ▸; Bonse, 1964 ▸; Chukhovskii & Petrashen’, 1977 ▸; Chukhovskii *et al.*, 1978 ▸; Kalman & Weissmann, 1983 ▸; Gronkowski & Malgrange, 1984 ▸; Chukhovskii & Malgrange, 1989 ▸; Gronkowski, 1991 ▸; Honkanen *et al.*, 2018 ▸).

Recently, hard X-ray free-electron lasers (XFELs) have gone into operation around the world (Emma *et al.*, 2010 ▸; Ishikawa *et al.*, 2012 ▸; Milne *et al.*, 2017 ▸; Kang *et al.*, 2017 ▸; Weise & Decking, 2018 ▸). At all of these sources, XFEL pulses originate from random current fluctuations in the electron bunch (Saldin *et al.*, 2000 ▸), which give rise to an individual time structure and energy of each pulse. The energy spectra of single pulses need to be characterized in a non-invasive way, allowing further use of the same pulses in the experiments.

Two basic requirements for the spectrometers – the acceptance range of photon energy and the energy resolution – follow from the duration of the pulse and the duration of the spikes in it (Saldin *et al.*, 2000 ▸). A spike duration of 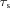 = 0.1 fs gives rise to an energy range that needs to be covered by the spectrometer 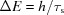 = 40 eV, where *h* = 4.13 eV fs is the Planck constant. When an X-ray beam of a width *w* is incident on a crystal bent to a radius *R*, the range of available Bragg angles 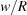 has to exceed the required angular range 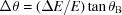, where 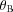 is the Bragg angle. Taking 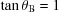 for simplicity and *E* = 12 keV as a reference energy, we find that, for a beam of width *w* = 500 µm, the curvature radius should be less than *R* = 15 cm to cover the whole spectrum. The bending radii of 5 cm for a 10 µm-thick silicon crystal (Zhu *et al.*, 2012 ▸) and 6 cm for a 20 µm-thick diamond (Boesenberg *et al.*, 2017 ▸) are reached. The resolution requirement for a spectrometer follows from the total duration of a pulse up to 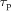 = 50 fs, which gives 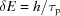 = 0.08 eV.

Different types of spectrometers based on silicon crystals have been proposed, built and tested for this purpose. They employ a focusing mirror with a flat diffracting crystal (Yabashi *et al.*, 2006 ▸; Inubushi *et al.*, 2012 ▸), a focusing grating (Karvinen *et al.*, 2012 ▸), a bent diffracting crystal (Zhu *et al.*, 2012 ▸) and a flat grating with a bent diffracting crystal (Makita *et al.*, 2015 ▸). A spectrometer based on beam focusing by compound refractive lenses with a flat diffracting crystal as dispersive element was proposed and analysed theoretically (Kohn *et al.*, 2013 ▸).

Recently, a spectrometer based on a bent thin diamond crystal has been designed and tested (Boesenberg *et al.*, 2017 ▸; Samoylova *et al.*, 2019 ▸) for high-repetition-rate XFEL sources, such as the European XFEL and LCLS II. Diamond is the material of choice for high-repetition-rate XFELs because only diamond can sustain the enormous peak heat load and prevent severe vibrations when the thermal stress wave is excited under repeated heat load in the megahertz range at a resonant frequency of the thin crystal plate.

The studies of XFEL pulses using diffraction on bent crystals (Zhu *et al.*, 2012 ▸; Makita *et al.*, 2015 ▸; Boesenberg *et al.*, 2017 ▸; Rehanek *et al.*, 2017 ▸) treated diffraction purely geometrically, as a mirror reflection of a geometric ray at a point where it meets the crystal surface. The process of diffraction in the crystal has not been taken into account, despite crystal thicknesses of 10 to 20 µm, which exceed the extinction lengths of dynamical diffraction for respective reflections (see estimates in the next section).

The studies of dynamical diffraction on bent crystals cited above considered the bending of thick crystals to radii varying from hundreds of metres to single metres. The curvature radius of some hundreds of metres already provides detectable broadening of the Darwin rocking curve, while bending to a radius of 1 m strongly modifies it. The results of these studies are not applicable to the case under consideration, where the crystal is thin and the bending radius is much smaller.

In the present paper, we consider X-ray diffraction on crystals bent to a radius of 10 cm or less. In the case of such strong bending, the incident X-ray wave remains at diffraction conditions (*i.e.* within the Darwin width of the actual reflection) only when propagating through distances that are small compared with the extinction length. As a result, back-scattering of the diffracted wave to the transmitted one is minor and diffraction is kinematical. We calculate diffraction from a bent crystal in both dynamical and kinematical theories and establish the applicability criterion for the approximation of kinematical diffraction.

We obtain a displacement field in the bent crystal by considering cylindrical bending of an elastically anisotropic rectangular thin plate by two momenta applied to its orthogonal edges. We show that, for a 110-oriented diamond plate, the elastic constants of diamond give rise to a very small strain variation along the plate normal because the Poisson effect on bending is almost completely compensated by the effect of anisotropy. As a result, the resolution of a bent crystal spectrometer is limited by the crystal thickness and can be better than the resolution of a non-bent crystal, limited by the extinction length.

We simulate XFEL spectra after diffraction on a bent crystal and show that an energy resolution of 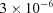, or 0.04 eV for the X-ray energy of 12 keV, can be reached on diffraction on a 20 µm-thick diamond crystal bent to a radius of 10 cm. We also take into account the free-space propagation of the waves diffracted by the bent crystal to the detector (Fresnel diffraction) and describe modifications of the spectra due to a finite distance to the detector.

## 2. Dynamical versus kinematical diffracted intensities   

For numerical estimates in this section, we consider, as a reference example, the symmetric Bragg reflection 440 of X-rays with energy *E* = 12 keV (wavelength λ = 1.03 Å) from a *D* = 20 µm-thick diamond crystal bent to a radius *R* = 10 cm.

When the crystal is not bent and oriented to satisfy the exact Bragg condition in symmetric reflection geometry, penetration of an X-ray wave in it is governed by the extinction length Λ, defined as a depth at which the amplitude of the wave decreases by a factor of *e* (correspondingly, intensity decreases 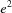 times). The extinction length is equal to 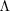 = 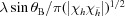, where 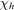 and 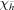 are the Fourier components of crystal susceptibility. For our example, the extinction length amounts to (Stepanov, 2004 ▸, 2019 ▸) Λ = 13.6 µm. The crystal thickness in our reference example is larger than the extinction length, and hence diffraction in a non-bent crystal should be calculated in the framework of dynamical diffraction theory.

Dynamical diffraction (strong coupling between the transmitted and the diffracted waves) takes place as long as the lattice distortions (the lattice spacing and the orientation of lattice planes) do not change on the distance Λ, or the change is much less than the width of the Darwin curve 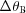 = 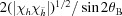, which in our case is 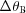 = 4.2 µrad (Stepanov, 2004 ▸, 2019 ▸). For a bent crystal of radius *R*, the gradient of distortions is 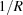 and its change on the distance of the extinction length is 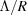. If the crystal is so strongly bent that this change is much larger than 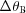, dynamical diffraction effects become negligible, since the path of the transmitted wave under diffraction conditions is much less than the extinction length. Such an estimate is similar to the treatment of the interbranch scattering in the vicinity of crystal lattice defects by Authier & Balibar (1970 ▸) and Authier *et al.* (1970 ▸) and predicts that the dynamical diffraction effects can be neglected for bending radii 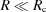, where 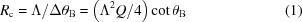
is a critical radius. Here 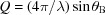 is the diffraction vector. For our example, 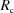 = 3.2 m.

To verify the applicability of the approximation of kinematical diffraction, we perform calculations of Bragg diffraction from a bent crystal plate in both dynamical and kinematical diffraction theories. In the calculations, the Fourier component of susceptibility 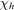 can be varied arbitrarily. The kinematical scattering amplitude is proportional to 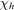 (and hence intensity is proportional to 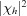) for any fixed bending radius, while the dynamical scattering amplitude depends on both 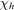 and *R* in a complicated way. Hence, the applicability of the kinematical theory can be established in the framework of dynamical diffraction, by studying the dependence of the diffracted intensity on 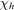. This is done in the present section. In the next section, we directly compare the kinematical and the dynamical scattering intensities.

Dynamical diffraction is calculated by numerical solution of the Takagi–Taupin equations (Takagi, 1962 ▸, 1969 ▸; Taupin, 1964 ▸):
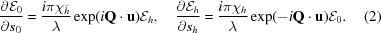
Here 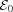 and 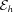 are the amplitudes of the transmitted and the diffracted waves, respectively, 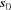 and 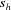 are the coordinates in the propagation directions of these waves, 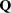 is the scattering vector and 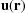 is the displacement vector. It describes the displacement of atoms from their positions in a reference non-bent crystal. The displacement 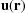 changes the susceptibility 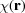 of the reference crystal to 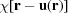, and Fourier expansion of the susceptibility over reciprocal-lattice vectors 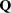 gives rise to the terms 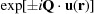 in equations (2). The algorithm of numerical solution of equations (2) was proposed by Authier *et al.* (1968 ▸) and revisited later by Gronkowski (1991 ▸) and Shabalin *et al.* (2017 ▸). To proceed to numerical solution of the Takagi–Taupin equations, we specify first the diffraction geometry and the displacement field 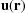 entering these equations.

**Figure 1:**
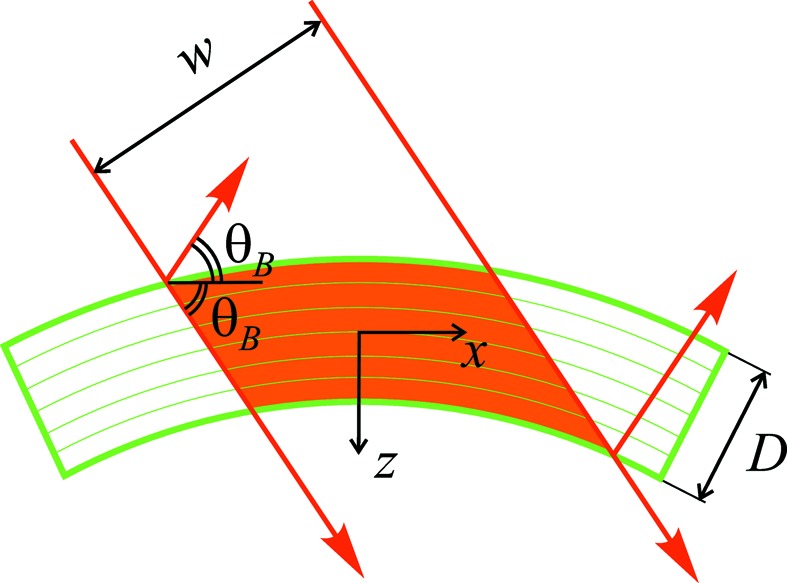
Geometry of symmetric Bragg diffraction from a bent crystal.

Fig. 1 ▸ sketches symmetric Bragg diffraction from a bent crystal plate. The scattering plane is the *xz* plane and the crystal is bent about the *y* axis. An ultrashort XFEL pulse, represented by its energy spectrum, is a coherent superposition of the waves with the same propagation direction and different wavelengths. We take a reference wavelength in the middle of the pulse spectrum and choose the origin 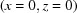 at a point in the middle plane of the crystal plate where the incident and the diffracted waves of the reference wavelength make the same angle 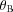 with the lattice planes.

The incident beam is restricted by a width *w*. The width of the wavefront of an XFEL pulse at the experiment is about 1 mm, much larger than the crystal thickness, but it can be focused to tens of microns, comparable with the crystal thickness. The estimate below shows that, if the beam is not focused, its width is much larger than the width of the diffracting region of the strongly bent crystal. The outer parts of the beam occur out of Bragg diffraction, and hence the beam width does not restrict diffraction.

Besides a focused incident beam, the width of the incident beam becomes essential when the bent crystal is rotated to measure its rocking curve (Samoylova *et al.*, 2019 ▸). The diffracted intensity decreases when the crystal is rotated such that the region of the crystal oriented at the Bragg angle to the incident beam goes out of the illuminated region of the crystal. This is reached for the angular deviations from the Bragg angle 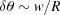. Hence, the width of the rocking curve of a bent crystal is given by the width of the incident beam. In all other situations, *i.e.* if the incident beam is not focused to a few tens of microns at the crystal and the angular deviation of the crystal is small compared with its rocking-curve width, the width of the incident beam is irrelevant. In the practical case, we take *w* = 500 µm in the calculations below and ensure that the diffracted intensity does not change with a further increase of the beam width.

In symmetric Bragg-case diffraction considered here, the diffraction vector 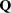 is in the negative direction of the *z* axis and 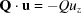, so that only the *z* component of the displacement vector in the bent crystal is of interest. It is calculated in Appendix *A* [App appa] taking into account the elastic anisotropy of a crystal with cubic symmetry. The displacement field in a crystal cylindrically bent to a radius *R* can be written as [*cf*. equation (29)] 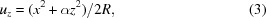
where the constant α depends on the elastic moduli and the crystal orientation [see equation (30)]. The elastic moduli of diamond give rise to exceptionally small values of α: we find α = 0.02 for a 110-oriented plate bent about the 001 axis and α = 0.047 for a 111-oriented plate bent about the 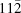 axis. For comparison, the elastic moduli of silicon result in α = 0.18 and 0.22 for these two orientations, respectively.

**Figure 2:**
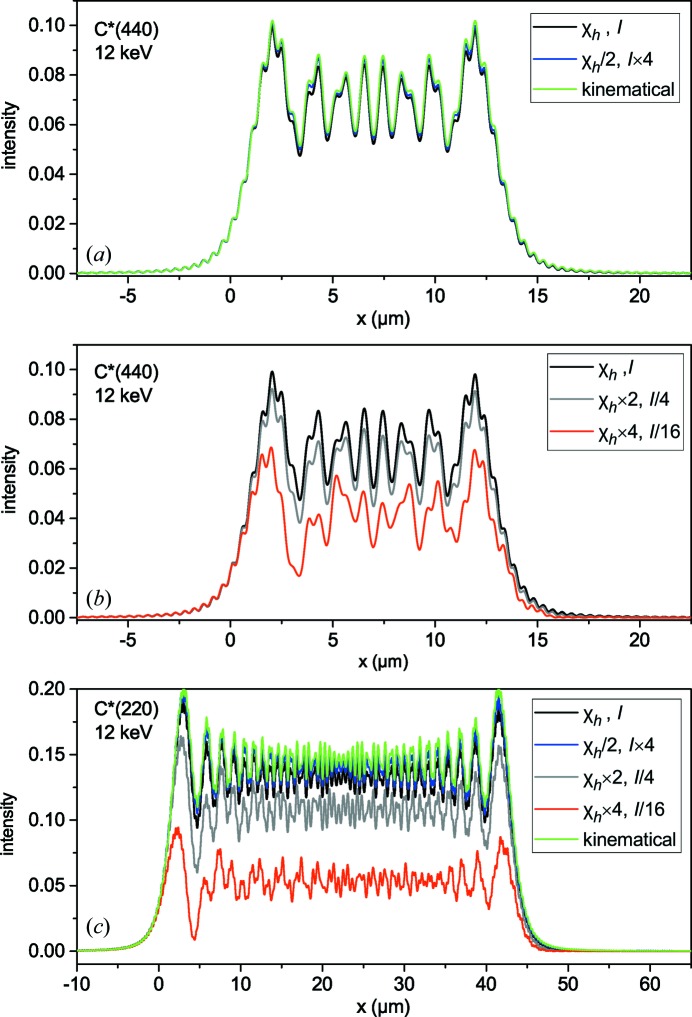
Dynamical and kinematical intensities of a diffracted wave at the crystal surface in symmetric Bragg reflections (*a*), (*b*) 440 and (*c*) 220 from a 20 µm-thick diamond crystal bent to a radius of 10 cm. The X-ray energy is 12 keV. Dynamical diffraction calculations for the X-ray susceptibilities 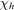 of the respective reflections (black lines) are repeated taking susceptibility smaller by a factor of 2, with the intensity multiplied by a factor of 4 (blue lines). Dynamical diffraction calculations are also performed with the susceptibilities 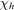 multiplied by factors 2 and 4, and with the respective intensities divided by factors 4 and 16 (grey and red lines). The kinematical intensities calculated by equation (7) are shown by green lines.

Fig. 2 ▸(*a*) shows by the black line the intensity distribution of the dynamically diffracted wave at the crystal surface for our example case. The spatial width of the diffracted wave is much smaller than the width of the incident wave and is determined by the crystal thickness projected to the surface at the Bragg angle. The amplitude of the incident wave is taken equal to 1. The amplitude of the diffracted wave is small compared with it, which points to kinematical diffraction.

Kinematical diffraction at the bent crystal simplifies theoretical analysis below. It is advantageous also from the experimental point of view, since it reduces a distortion of the X-ray pulse passing through the bent crystal spectrometer and intended to be used further in an experiment.

To verify the kinematical nature of diffraction further, we perform the same calculation but, instead of the susceptibility 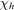, use the value 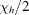 without changing any other parameter. When the approximation of kinematical diffraction is applicable, the diffracted amplitude is expected to be proportional to 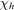, so that the intensity is proportional to 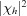. Hence, we multiply the calculated intensity by a factor of 4 (blue line) and compare with the former calculation with the initial value 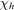 (black line). The curves practically coincide, which further shows the kinematical nature of diffraction. Thus, Fig. 2 ▸(*a*) demonstrates, by means of the calculations made in the framework of dynamical theory, the applicability of the approximation of kinematical diffraction for curvature radii small compared with the critical radius (1).

In Fig. 2 ▸(*b*), we calculate dynamical diffraction intensity in the same reflection but with the susceptibility 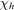 increased by factors 2 and 4, with the aim of establishing the applicability limits of the approximation of kinematical diffraction. Since the critical radius 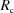 in equation (1) is proportional to 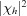, the increase of 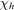 by a factor of 2 reduces the critical radius from 3.2 m to 80 cm, still large compared with the bending radius of 10 cm. The calculated curve [grey line in Fig. 2 ▸(*b*)] deviates from the reference curve (black line) mostly by a scale factor. When the susceptibility 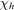 is increased by a factor of 4, and hence the critical radius reduced to 20 cm, the calculated diffraction intensity (red curve) notably differs from the reference black curve not only in scale but also in the shape of fringes. Thus, approaching the critical radius (1) results in a strong modification of the diffracted intensity.

Fig. 2 ▸(*c*) collects similar calculations for the C*(220) reflection under the same conditions. For this reflection of 12 keV X-rays, the Bragg-case extinction length and the Darwin width are (Stepanov, 2004 ▸, 2019 ▸) Λ = 4.17 µm and 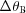 = 8.63 µrad, so that the critical radius 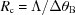 = 48 cm, and the bending radius of 10 cm occurs closer to the critical radius. Calculations with the susceptibility 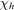 for this reflection (black line) and for two times smaller susceptibility (blue line) differ slightly by a scale factor, so that the approximation of kinematical diffraction is applicable but close to its applicability border. When the susceptibility is increased by a factor of 2 (grey line), the critical radius becomes 12 cm, close to the bending radius. The calculated diffraction intensity notably differs from the reference black curve. When the susceptibility is increased by a factor of 4 and the critical radius becomes as small as 3 cm, the fringes of the calculated intensity (red curve) do not follow the reference curve, again confirming that, for radii smaller than the critical radius (1), the use of dynamical theory is necessary.

**Figure 3:**
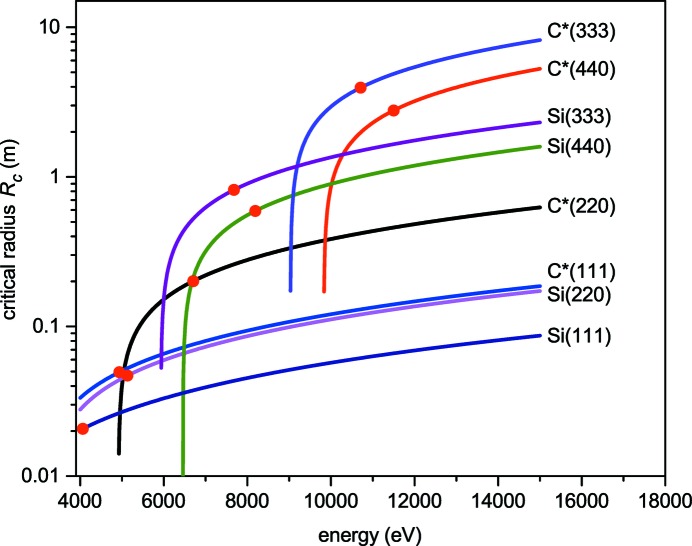
The X-ray energy dependence of the critical radii given by equation (1) for several reflections of diamond and silicon. The point at each curve marks an energy such that, for a crystal thickness 20 µm and distance to detector 1 m, the width of the beam diffracted from the bent crystal is equal to the width of the first Fresnel zone. The Fraunhofer approximation is applicable, under these conditions, for energies smaller than the marked energy (see Section 5 for details).

The analysis in the next sections shows that the applicability of the approximation of kinematical diffraction not only simplifies calculation of the intensity diffracted by the bent crystal but leads to a resolution better than that given by the Darwin width of dynamical diffraction. Therefore, the critical radii for different reflections are of interest. Fig. 3 ▸ presents critical radii for symmetric Bragg reflections from diamond and silicon crystals as a function of the X-ray energy. Since the energy range presented in Fig. 3 ▸ is far from the absorption edges of carbon or silicon, the susceptibilities 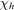 are proportional to 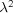. Then, the extinction length does not depend on λ and, as follows from the second equality in equation (1), the energy dependence of the critical radius is simply given by the factor 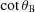.

Thus, in this section, we have verified, entirely by means of calculations performed in the framework of dynamical theory, the criterion (1) for applicability of the approximation of kinematical diffraction. In the next section, we calculate the kinematical amplitude and compare it with the calculations of dynamical diffraction.

## 3. Kinematical diffraction amplitude at the crystal surface and in the far field   

### 3.1. Amplitude at the crystal surface   

The kinematical diffraction amplitude at the crystal surface 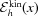 can be obtained by neglecting the influence of the diffracted wave 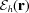 on the transmitted wave 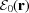 in the first Takagi–Taupin equation (2). Then the amplitude of the transmitted wave in the crystal is given by the first equation shortened to 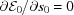, which gives 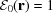. The diffracted wave is determined by the solution of the second equation, which becomes now
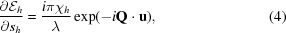
with the boundary condition 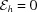 at the bottom surface of the crystal 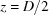. To simplify calculations, we restrict ourselves in this section to the case of a 110-oriented diamond crystal with its very small value of α, and take 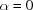 in equation (3). The general form of the kinematical integral is introduced and studied in Section 4. The amplitude of the diffracted wave at the top surface 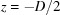 is 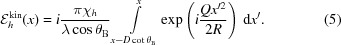
The integration range in equation (5) corresponds to the integration along the direction of the diffracted wave, making an angle 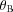 with the *x* axis, from the bottom to the top surface of the crystal. Since the integrand in equation (5) does not depend on *z*, the integration along 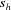 is replaced with the integration over 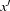 by 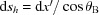.

The integral (5) can also be written, by substituting 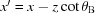, as an integral over crystal thickness, 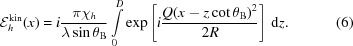
Calculation of the integral is straightforward, 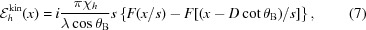
where 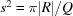, and it is denoted 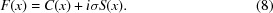
Here 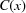 and 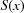 are cosine and sine Fresnel integrals, 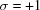 for 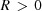 (convex surface of the bent crystal, as shown in Fig. 1 ▸) and 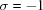 for 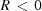 (concave crystal surface).

Green lines in Figs. 2 ▸(*a*) and 2 ▸(*c*) show kinematical intensity 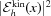 calculated with the same values of all parameters as in the corresponding dynamical diffraction calculations. The kinematical intensity almost coincides with the dynamical one, thus providing a final proof for the applicability of the approximation of kinematical diffraction for curvature radii that are small compared with the critical radius. We note that the coincidence of the curves is reached on the absolute scale, without adjusting intensities.

### 3.2. Fraunhofer diffraction   

The diffracted wave at the crystal surface 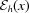 transforms during further propagation of the wave in free space to a detector. At large enough distances from the diffracting crystal (Fraunhofer diffraction), the X-ray wavefield is described by the Fourier transform of 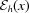. Let us consider the field distribution at such distances, assuming that the field transformation in the *y* direction normal to the scattering plane is still not involved. Transformation of the wave diffracted by the bent crystal on propagation in free space over finite distances (Fresnel diffraction) is considered in Section 5.

To obtain the Fourier spectrum of the kinematical diffraction amplitude (5), we represent it as a convolution integral,
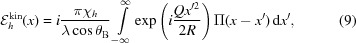
where the function 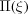 is defined as 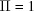 for 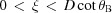 and 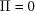 out of this interval. Making the Fourier transformation of the two terms under the integral, we get 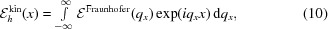
where 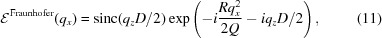


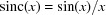
, 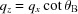, and a constant prefactor is omitted in equation (11) to simplify expressions. Intensity in the far field (Fraunhofer diffraction) is given simply by 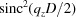, which provides a resolution inversely proportional to the thickness *D*. Under conditions of kinematical diffraction, it can be better than the resolution of dynamical diffraction, which is limited by the extinction length. This resolution is studied further in the next section.

## 4. Spectral resolution   

Equations in the previous section do not include an angular deviation of the incident wave from the Bragg condition and are restricted with the limit 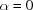. To avoid these restrictions and also allow a coherent superposition of waves with different wavelengths, we use a more general expression for the kinematical diffraction amplitude as an integral over the scattering plane of the crystal,
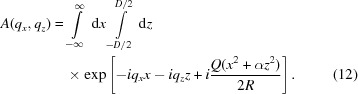
We restrict ourselves in this section to the Fraunhofer diffraction. The wavevector 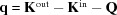 is the deviation of the scattering vector 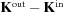 from the reciprocal-lattice vector 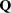. We have 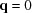 for the wave of the reference wavelength incident on the crystal exactly at the Bragg angle 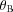 corresponding to that wavelength and reflected at the Bragg angle. The components of the scattering vector 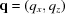 depend on the angular deviations 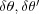 of both incident and scattered waves, and on the deviation 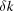 of the length of the wavevector in the incident spectrum from the reference wavevector 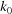 (since scattering is elastic, the lengths of the wavevectors of the incident and the scattered waves coincide). Explicit expressions for 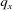 and 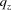 are derived in Appendix *B* [App appb]. It is convenient, for the purpose of comparison of the incident and the diffracted spectra of an XFEL pulse, to represent the diffracted intensity in an energy spectrum by considering the scattering angle 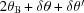 as a Bragg angle for the respective wavevector 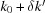. The components 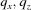 of the scattering vector expressed through the angular deviation of the incident beam 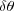 and the wavevector deviations 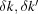 are given by equation (40). In particular, an XFEL pulse can be described as a coherent superposition of plane waves with different wavelengths propagating in the same direction. With the crystal oriented at the Bragg angle for the reference wavelength (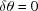), we get 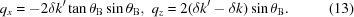



The *x*-dependent terms of the phase in the integral (12) can be recollected as 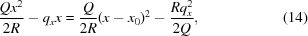
where 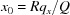. The exponential factor in the integral with this phase oscillates strongly everywhere except an interval of the width 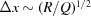 around a point 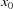. This range of *x* provides the main contribution to the integral. For a monochromatic wave with an angular deviation 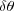 from the Bragg orientation, we get from equation (39) that the centre of the diffracting region occurs at 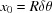. When the angular deviation of the incident wave is so strong that 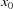 exceeds the width of the incident wave, the interval of *x* contributing to diffraction goes out of the illuminated part of the crystal, which causes a decrease of the diffracted intensity and defines the rocking-curve width of the bent crystal. For smaller angular deviations, the interval 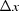 is within the illuminated area, and the width *w* does not restrict diffraction. In Appendix *C* [App appc], we explicitly calculate the kinematical integral (12) for a Gaussian profile of the incident wave with a width *w*, as sketched in Fig. 1 ▸. The resulting expression is rather bulky. In most cases of practical interest, the width of the incident beam is so large that the outer parts of the beam are out of diffraction, and the width *w* does not limit diffraction. We consider this latter case further on.

The range 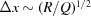 of the diffracting region in the bent crystal increases with increasing curvature radius *R*. However, the applicability of the kinematical approximation is limited by the curvature radii 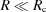. Using the second expression for 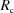 in equation (1), we find that 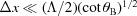. We do not consider very small Bragg angles and conclude that the range of *x* contributing to the integral (12) is much smaller than the extinction length Λ. This result provides an additional insight into the origin of the kinematical diffraction in bent crystals: as long as the condition (1) of kinematical diffraction is satisfied, the diffraction takes place in a narrow column of width 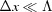 in the crystal. The diffracted wave leaves this column and cannot influence the transmitted wave, even when the thickness exceeds the extinction length.

The kinematical integral (12) splits into a product of two integrals, one over *x* and the other over *z*. Since we consider the region 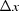 to be within the illuminated area, the integral over *x* is calculated in infinite limits. The remaining integral is over *z*, 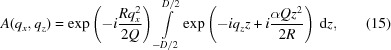
where we again omit a constant prefactor. When 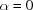, equation (15) reduces to equation (11) but allows for more general expressions (40) for the components of the vector 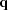.

**Figure 4:**
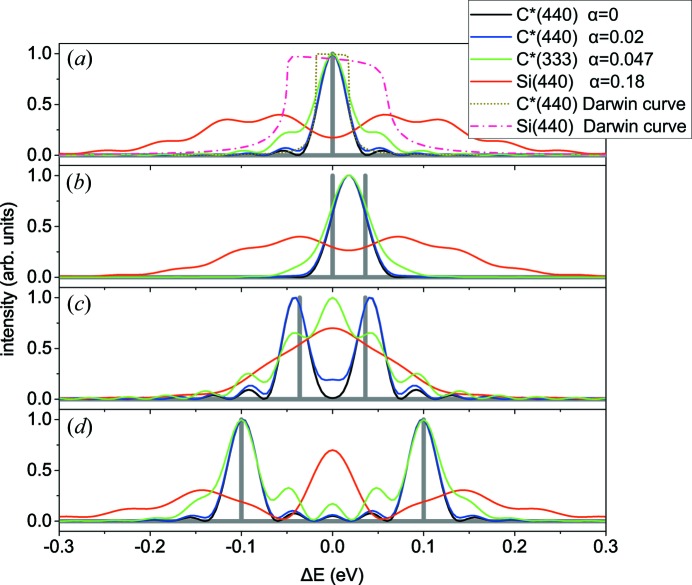
Angular distributions of the waves diffracted by 20 µm-thick crystal plates bent to a radius of 10 cm, calculated by equation (18) and represented in the same energy spectrum as the incident waves. The incident spectra are shown by thick grey lines and consist of (*a*) a single monochromatic plane wave of energy 12 keV or (*b*)–(*d*) two coherent monochromatic waves with small differences in wavelengths. The dotted lines in (*a*) show the Darwin rocking curves for C*(440) and Si(440) reflections.

Let us focus first on this limiting case 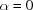, which is of special interest since it corresponds to the case of the 110-oriented diamond plate. In this case, the scattering intensity due to an incident monochromatic plane wave is simply 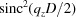. The intensity distribution is the same as in the classical problem of diffraction grating in light optics. It is shown in Fig. 4 ▸(*a*) by a black line.

The dotted line in Fig. 4 ▸ *(a*) is the Darwin rocking curve from a non-bent semi-infinite crystal in the same symmetric Bragg reflection C*(440). Its full width at half-maximum is close to that of a bent 20 µm-thick crystal. A thicker bent crystal will provide a narrower curve. We note that its width does not depend on the bending radius.

Fig. 4 ▸(*a*) also presents the angular distribution of the waves diffracted from a 20 µm-thick silicon crystal, bent to the same radius of 10 cm, in the same reflection 440, and the Darwin curve for this reflection. Both curves are several times broader than the respective curves in the C*(440) reflection, but the reasons for their broadening are different. A broader Darwin curve results from a larger susceptibility and a smaller Bragg angle of the Si(440) reflection with respect to the C*(440) reflection. The width of the angular distribution of the waves diffracted by the bent crystal does not depend on the susceptibility, because of the kinematical diffraction, and the broader curve in the Si(440) reflection is due to a larger value of the parameter α.

The possibility of resolving two waves with the same incidence direction and different wavelengths is commonly defined in light optics by the Rayleigh criterion (two wavelengths are resolved if the maximum of diffracted intensity from one of them corresponds to the first minimum of the other). In the case 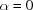 this criterion, applied to the sum of intensities 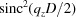 for two different wavelengths, gives the resolution 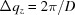, and hence 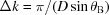. The resolution is limited by the crystal thickness, which can be larger than the extinction length. Hence, kinematical diffraction on a strongly bent crystal can provide better resolution than the dynamical diffraction on a planar crystal. The analysis above explains this surprising result: kinematical diffraction on a strongly bent crystal takes place in a column whose width 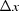 is small compared with the extinction length, but (for 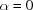) the height is equal to the crystal thickness *D*. That results in a kinematical scattering from a bent crystal whose thickness is not limited by the extinction length. The energy resolution 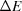 is related to the momentum resolution 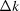 simply by 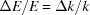, so that the Rayleigh criterion reads 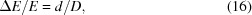
where *d* is the interplanar distance of the actual reflection and the Bragg law 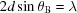 is used. For our example case, we get 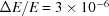 and 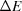 = 0.04 eV. The latter value is close to the width of the Darwin curve for a semi-infinite non-bent crystal [see Fig. 4 ▸(*a*)].

Equation (16) is applicable, theoretically, as long as the thickness of the crystal remains small compared with the X-ray absorption length, since absorption is neglected in the analysis above. Practically, however, the crystal thickness is limited by much smaller values: thick plates do not suffer bending to small radii.

Boesenberg *et al.* (2017 ▸) considered diffraction on a bent crystal purely geometrically and arrived at a resolution defined by the pixel size of a detector. Its contribution can be added to the diffraction-limited resolution (16), when needed.

The energy spectrum of an XFEL pulse originates from the spectral expansion of a short pulse, so that different wavelengths contribute coherently and the amplitude 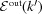 of the electric field of the diffracted wave is related to the amplitude 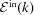 of the electric field incident on the bent crystal by 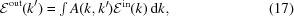
where the diffraction amplitude 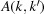 is described by equations (18) or (43) with the components of the wavevector 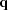 given by equation (13).

Fig. 4 ▸(*b*) presents angular distributions of the waves diffracted by a bent crystal when the incident wave is a coherent superposition of two monochromatic waves with the wavelength difference corresponding to the Rayleigh criterion (16). The angular distributions are represented as corresponding spectra, as described above. The two monochromatic components are not resolved since the Rayleigh criterion is formulated for two incoherent waves and implies the sum of intensities, rather than the sum of amplitudes.

Fig. 4 ▸(*c*) shows calculated angular distributions of the diffracted waves for the wavelength difference between two coherent monochromatic components two times larger than given by the Rayleigh criterion. The components are well resolved. The resolution, defined as the ability to resolve two monochromatic lines, in the case of the coherent superposition of two waves is about 1.5 times worse than that given by the Rayleigh criterion (16). Fig. 4 ▸(*d*) shows calculated spectra for a larger wavelength difference of the two monochromatic components of the incident wave. The components are well resolved for 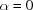 (black line).

The resolution (16) is obtained by neglecting the second term in the exponent in the integral (15). This is possible as long as α is so small that 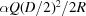 is much smaller than 1. In the general case 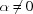, calculation of the integral gives 
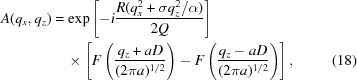
where 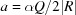 and the function 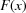 is defined in equation (8).

For the C*(440) reflection, the factor 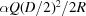 is approximately equal to 1, and the calculated curves (blue curves in Fig. 4 ▸) are close to the calculation with 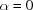 (black curves). This factor calculated for the C*(333) reflection (with 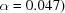 is approximately equal to 2, which already results in a notable modification of the diffraction curves (green curves in Fig. 4 ▸). For the Si(440) reflection with 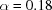, this factor is 5.9, which results in complicated diffraction patterns (red curves in Fig. 4 ▸), rather than a broadening of the corresponding spectral lines.

Fig. 4 ▸ shows that, due to a coherent superposition of the monochromatic components, the worse resolution for 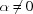 cannot be described as the broadening of the sharp peaks of the incident spectrum. Rather, a complicated interference pattern arises, and the incident spectrum can hardly be recognized in it. The width of the interference fringes is still given by equation (16).

## 5. Fresnel diffraction   

In this section, we consider the finite-distance free-space propagation of the wave diffracted by a bent crystal. This allows us to establish the applicability limits of the Fraunhofer approximation used in the previous section and evaluate corrections due to a finite distance from the bent crystal to a detector.

Let us follow the free-space propagation of the electric field at the crystal surface 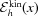 given by equations (5)–(7) for the case 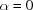. At a distance *L* from the bent crystal, the free-space propagation is described [see *e.g.* Born & Wolf (1964 ▸), Section 8.3, and Cowley (1975 ▸), Section 1.7] by multiplying the electric field at the crystal surface 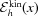 with the phase factor 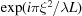, where ξ is the distance in the direction perpendicular to the propagation direction of the diffracted beam, 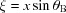: 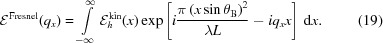
Substituting here equation (6) and performing integration over *x*, we represent equation (19) as 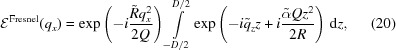
where we define 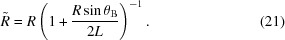
The quantities 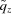 = 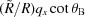 and 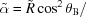 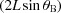 are introduced here for the particular case α = 0. Below in equation (24) they are derived for the general case 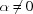. In the limit 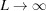, the Fresnel diffraction amplitude (20) reduces to the Fraunhofer one (11).

The distance *L* required to reach the Fraunhofer limit follows from equations (20) and (21). The first requirement is 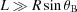, which gives 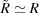. Since equation (20) is written for 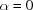, the second requirement follows from the possibility of neglecting the second term in the exponent in the integral (20). This term at 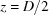 is equal to 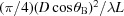. We note that the crystal thickness *D* seen from the direction of the diffracted beam is 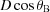, while the diameter of the first Fresnel zone is 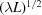. Hence, the crystal thickness seen from the direction of the diffracted beam should be smaller than the diameter of the first Fresnel zone, *i.e.* the distances from crystal to detector should be 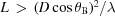. The minimum distance depends on the Bragg angle: for our reference case of the C*(440) reflection at 12 keV and crystal thickness *D* = 20 µm, we get *L* > 1.3 m, while, for C*(220) under the same conditions, we have *L* > 3.2 m. The points in Fig. 3 ▸ mark, for each reflection, the energy given by the condition 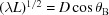 for the crystal thickness *D* = 20 µm and distance to detector *L* = 1 m. For energies smaller than marked, Fraunhofer approximation is approached at 1 m distance to the detector. Larger energies correspond to Fresnel diffraction at such a distance.

Calculation of the integral (20) gives
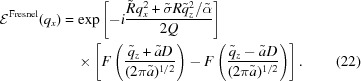
Since the bending radius *R* can be positive (convex surface of the bent crystal) or negative (concave crystal surface), we define a positive quantity 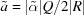 and the sign term 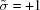 if 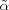 and *R* are of the same sign and 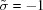 if 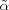 and *R* have opposite signs. The function 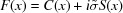 is defined similarly to equation (8).

**Figure 5:**
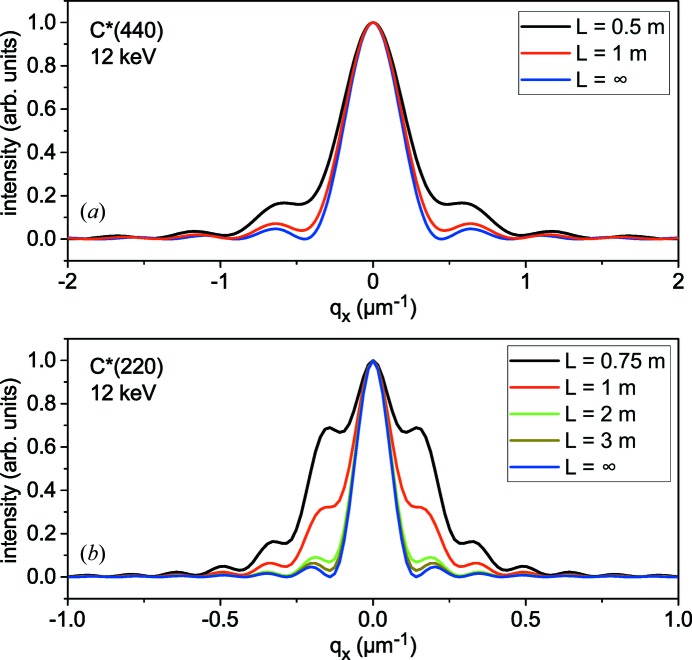
Transformation on the way to the detector of a monochromatic wave diffracted from a 20 µm-thick diamond crystal bent to a radius of 10 cm, calculated by equation (22). Reflections (*a*) 440 and (*b*) 220 are compared.

Fig. 5 ▸ shows transformation of the diffracted beam with the distance to the detector, calculated by equation (22). Reflections 440 and 220 from diamond at the same energy 12 keV are compared. The only essential difference between reflections is their Bragg angles: the larger Bragg angle of the 440 reflection gives rise to smaller distances needed to reach the Fraunhofer diffraction range.

In the analysis above, we used the amplitude of the wave diffracted by a bent crystal 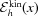 that was written for a monochromatic incident wave, exact Bragg orientation of the incident wave and the special case 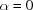. In the general case of the kinematical scattering amplitude (12), the free-space propagation is described by an additional phase term 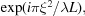
where 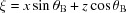 is the distance in the direction perpendicular to the beam diffracted by the crystal. Then, the amplitude of the diffracted wave at the detector is written as 
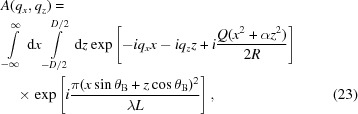
which replaces the respective integral (12) written for Fraunhofer diffraction. The integral (23) can be written in the same form as equation (20) with the same expression for 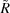 given by equation (21) but 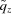 and 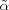 are generalized as follows: 
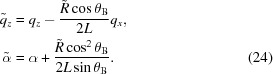
Calculation of the integral gives rise to equation (22). It has the same form as the Fraunhofer amplitude (18) but with the parameters modified according to equations (21) and (24).

We have already seen in the analysis of Fraunhofer diffraction in Section 4, and in particular in Fig. 4 ▸, that the value of parameter α plays an essential role in spectral resolution. Finite-distance free-space propagation of the wave diffracted from the bent crystal gives rise to a modification of this parameter to 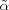, as given by equation (24). In particular, the concave bending (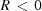) and appropriately chosen distance *L* can be used to reduce this parameter and hence improve the resolution.

**Figure 6:**
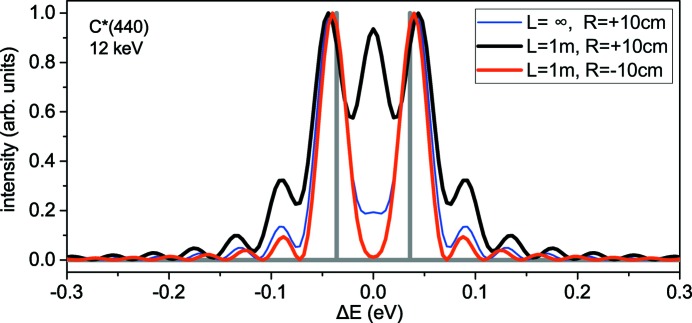
Spectra of diffracted waves for an incident wave consisting of two mutually coherent plane waves of different wavelengths (shown by thick grey lines) for an infinite distance to the detector (Fraunhofer diffraction, blue line) and the distance to detector *L* = 1 m (Fresnel diffraction), calculated by equation (22). Symmetric Bragg reflection 440 from a 20 µm-thick diamond plate, bending radius 10 cm, convex (black line) and concave (red line) bending are compared.

Fig. 6 ▸ shows calculated spectra of diffracted waves for an incident wave consisting of two coherent plane waves, the same as in Fig. 4 ▸(*c*). The blue line in Fig. 6 ▸ is calculated for an infinite distance *L* and represents the same line in Fig. 4 ▸(*c*). Black and red lines are calculated for a distance from the bent crystal to detector of *L* = 1 m. Calculation by equation (24) gives 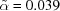 for a convex bending with *R* = +10 cm and 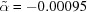 for a concave bending with *R* = −10 cm. The increase of 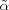 for the convex bending has the same effect as an increase in α for reflection C*(333) in Fig. 4 ▸ and gives rise to a more complicated spectrum with several fringes. The decrease of 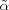 for the concave bending has an opposite effect and leads to a simple spectrum of two waves described by equation (11) with the resolution given by equation (16).

## 6. Spectra of XFEL pulses   

The spectra in the self-amplified spontaneous emission (SASE) mode of the European XFEL have been generated with the simulation code *FAST* (Saldin *et al.*, 1999 ▸), which provides a 2D distribution of electric field in real space at the exit of the undulator for each moment of time for various parameters of the electron bunch charge and the undulator. Simulation results are stored in an in-house database (Manetti *et al.*, 2019 ▸). The spectra are simulated for the electron energy 14 GeV, photon energy 12.4 keV, and the active undulator length corresponding to the saturation length, the point with the maximum brightness, for a given electron bunch charge (Schneidmiller & Yurkov, 2014 ▸).

Conversion from the time to the frequency domain has been performed using the *WavePropaGator* package (Samoylova *et al.*, 2016 ▸), which provides a 2D distribution of electric field for each frequency of the pulse. We use the spectrum at the centre of the pulse in frequency domain, assuming this distribution to be the same across the beam.

**Figure 7:**
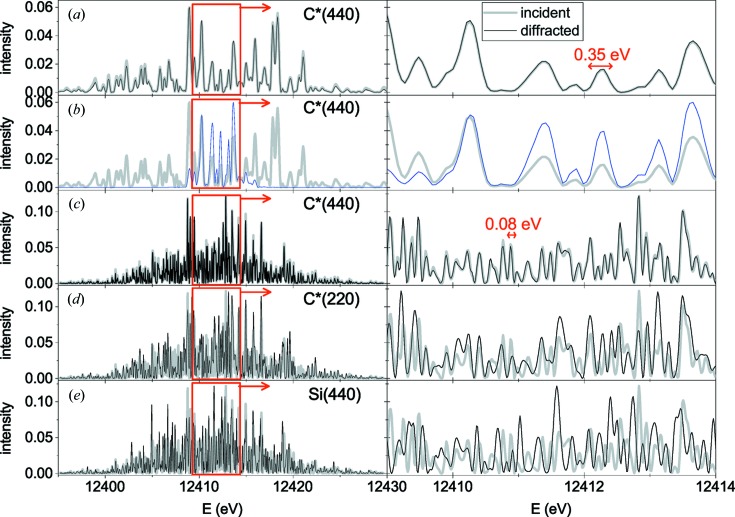
Spectra of the waves incident on (*a*)–(*d*) diamond or (*e*) a silicon plate of thickness *D* = 20 µm bent to a radius of *R* = 10 cm (thick grey lines) and spectra of the diffracted waves in the Fraunhofer diffraction case (thin black or blue lines). The incident beam width is *w* = 500 µm (*a*), (*c*)–(*e*) or 50 µm (*b*). The pulse duration is 10 fs (*a*), (*b*) and the undulator length is 75 m, or the pulse duration is 42 fs (*c*)–(*e*) and the undulator length is 105 m. The spectrum of the incident wave is convoluted, according to equation (17), with the scattering amplitude given by equations (18) for (*a*), (*c*)–(*e*) or (43) for (*b*).

Fig. 7 ▸ compares spectra of the XFEL pulses incident on the diffracting bent crystal (thick grey lines) and the spectra of the diffracted waves (thin black or blue lines). Complex amplitudes of the incident beams were used in the calculation of diffraction by equation (17); squared moduli of the amplitudes are shown in the figure and the respective phases are not shown. Calculations of the diffraction amplitude 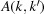 using equation (18) for an infinite width of the incident wave or using equation (43) taking into account the finite width of the incident beam give identical results for the width *w* = 500 µm in Figs. 7 ▸(*a*), 7 ▸(*c*)–7 ▸(*e*). For the width *w* = 50 µm of a focused beam in Fig. 7 ▸(*b*), equation (43) is used. The bending radius of the crystal is taken as *R* = 10 cm and its thickness *D* = 20 µm.

Figs. 7 ▸(*a*), 7 ▸(*b*) show by thick grey lines a spectrum of the XFEL pulse of the duration of approximately 10 fs generated in an undulator of active length 75 m. The pulse duration of 10 fs gives rise to a 0.35 eV characteristic width of the oscillations in the spectrum. The numbers above are the full width at half-maxima (FWHM) of the peaks in time and frequency domains, respectively. Such a spectrum is well resolved by the bent crystal spectrometer in the C*(440) reflection, as shown in Figs. 7 ▸(*a*), 7 ▸(*b*). A width of *w* = 500 µm of the incident beam is needed to resolve the whole spectrum [see Fig. 7 ▸(*a*)]. If the beam is focused to a width *w* = 50 µm, only a small part of the spectrum is diffracted [see Fig. 7 ▸(*b*)]. The characteristic width of the oscillations in the spectrum is still reproduced, and hence the pulse duration can be estimated.

Figs. 7 ▸(*c*)–7 ▸(*e*) show a spectrum of the X-ray pulse of duration 42 fs at the undulator length 105 m. This pulse duration gives rise to a 0.08 eV characteristic width of the oscillations in the spectrum. The resolution of the bent crystal spectrometer, estimated with the Rayleigh criterion (16), is about 0.04 eV. The continuous spectrum of the X-ray pulse is fully reproduced in the C*(440) reflection [see Fig. 7 ▸(*c*)]. The reflection C*(220), shown in Fig. 7 ▸(*d*), possesses, as follows from equation (16), two times worse resolution because of the two times larger interplanar distance *d*. The initial spectrum is not reproduced and its oscillations are not fully resolved. However, the oscillations are of almost the same width as in the initial spectrum. They can be used to estimate the pulse duration in the time domain with almost the same accuracy as the initial spectrum. In the reflection Si(440) presented in Fig. 7 ▸(*e*), the depth dependence of the displacement field due to the value of 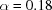 for silicon gives rise to a worse resolution. The initial spectrum is not reproduced but, as in the case of the C*(220) reflection, the oscillations can be used to estimate the pulse duration.

**Figure 8:**
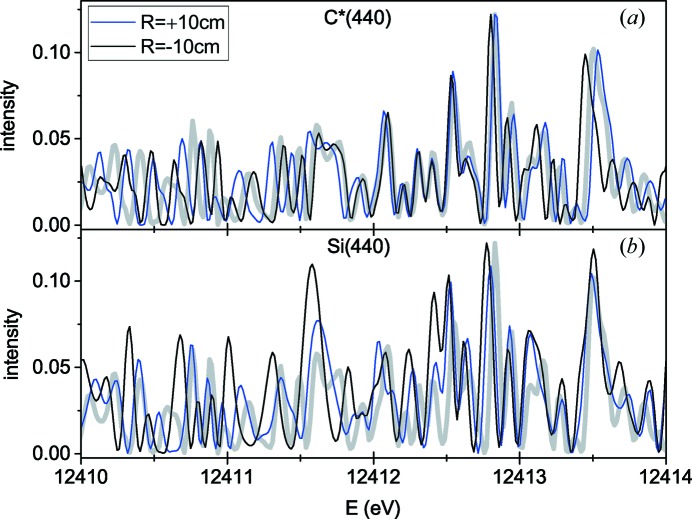
The incident (thick grey lines) and diffracted (thin black and blue lines) spectra at a distance *L* = 1 m from a 20 µm-thick diamond (*a*) or silicon (*b*) plate bent to a radius *R* = 10 cm. The spectrum of the incident wave is convoluted, according to equation (17), with the scattering amplitude given by equation (22).

Fig. 8 ▸ compares spectra calculated for a distance *L* = 1 m from the bent crystal to a detector, for C*(440) and Si(440) reflections for the same incident pulse as in Figs. 7 ▸(*c*)–7 ▸(*e*). Bending in opposite directions, concave and convex, is compared for each reflection. For the C*(440) reflection, the spectrum is somewhat expanded (at 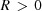) or compressed (at 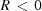) with respect to the spectrum of the incident pulse. For the Si(440) reflection, transformation of the spectrum is more complicated, but it does not change the structure of the spectrum qualitatively.

In all cases presented in Figs. 7 ▸ and 8 ▸, the spectra of the waves diffracted from a bent crystal are qualitatively similar to the spectra of the incident beams. The widths of the fringes in the spectra can be used to estimate duration of the incident pulses. However, only the C*(440) reflection reproduces the incident spectrum at the energy of 12 keV. Even in this case, the spectrum is slightly expanded or compressed, depending on the direction of bending, due to a finite distance from the bent crystal to the detector.

For other reflections, the spectra of the waves diffracted by the bent crystal do not coincide with the Fourier transformations of the incident pulses. However, when the conditions for kinematical diffraction are satisfied, they can be calculated for a given incident pulse using diffraction amplitudes derived above and used in a fitting procedure to obtain time structure of the incident pulse.

## 7. Conclusions   

X-ray diffraction from a bent single crystal can be treated kinematically when the bending radius is small compared with the critical radius given by the ratio of the Bragg-case extinction length for the actual reflection to the Darwin width of this reflection. The critical radius varies, depending on the X-ray energy, the crystal and the reflection chosen, from centimetres to metres.

Under conditions of kinematical diffraction, each monochromatic component of the pulse finds diffraction conditions only in a column inside the crystal with the width much smaller than the extinction length. In a cylindrically bent diamond plate of 110 orientation, the entire column diffracts in phase, since the Poisson effect on bending is compensated by the elastic anisotropy, and the displacement field does not vary over the depth. In this case, the spectral resolution is limited by the crystal thickness, rather than the extinction length, and can be better than the resolution of a planar dynamically diffracting crystal. It amounts to the ratio of the lattice spacing for the actual reflection to the crystal thickness. As an example, the symmetric Bragg reflection 440 from diamond provides an almost undistorted spectrum for X-ray energies of about 12 keV with the resolution of 0.04 eV.

The spectrum of the waves diffracted by the bent crystal generally differs from the spectrum of the incident pulse. Hence, the spectrum is not resolved in a rigorous spectroscopic sense. However, the diffracted spectra look qualitatively similar to the respective incident spectra. The widths of their fringes can still be used to estimate duration of the incident X-ray pulse. A finite distance from the bent crystal to a detector (Fresnel diffraction) causes additional modifications of the measured spectrum, but still leaves it qualitatively similar to the incident one.

## Appendix A Displacement field in a bent anisotropic thin plate

### A1. Elastic equilibrium equations and their solution

To calculate the displacement field in a bent plate, taking into account its elastic anisotropy, we begin with Hooke’s law in the  formulation , where  denote pairs of indices (, , , , , ). Here,  are the components of the compliance tensor, and the prime denotes the components in the coordinate system with the  axes in the plane of the plate and the *z* axis normal to it. The notation  without the prime is reserved for the components of the compliance tensor in the standard cubic reference frame. The components of the stress tensor are denoted by , and the components of the strain tensor  are written in the engineering notation (*i.e.* without the coefficient 1/2 at the off-diagonal components):

The absence of forces at the plate surface gives  (where ) and, since the plate is thin, these components of stress are small in comparison with the other stress components also inside the plate, so that .

We consider bending of the plate by two moments,  about the *y* axis and  about the *x* axis, which give rise to stress linearly varying across the plate [see Lekhnitskii (1981 ▸), equation (16.1)]:

where *D* is the plate thickness. We do not include torsion in the consideration and hence take . Thus, the components  and  in equation (26) are the only nonzero stress components, and the elastic equilibrium equations read

The solution of these equations, with the centre of the plate fixed at zero ( at the point ) is [see Lekhnitskii (1981 ▸), equation (16.3)]

For the symmetric Bragg-case diffraction considered in the present work, only the displacement normal to the plate plane  is of interest. Crystal orientations that we consider (see the next section) give  =  = 0. Then, the displacement for a biaxial bending can be written in the form  = . The curvature radii  and  are easily derived from equation (28), and we do not present these bulk expressions here. Rather, we consider the case of cylindrical bending, when the bending moments  and  are applied to provide . In this case, the displacement field can be written as

where

We omit a bulky expression of the curvature radius *R* through the bending moments, since the radius, rather than the moments, is directly measured in the experiment.

The aim of the next sections is to calculate the coefficient α for different crystallographic orientations of the plate and for different materials. For that purpose, the compliances  need to be calculated for the respective crystallographic orientations.

### A2. Transformation of the compliance tensor

Transformation of the components  from the reference coordinate system with the standard axes of the cubic crystal to the coordinate system related to the crystallographic orientation of the plate requires rotation of the fourth-rank tensor  to the new coordinate system by four rotation matrices. Wortman & Evans (1965 ▸) and Lekhnitskii (1981 ▸, Section 5) proposed two different practical methods to make this transformation. We did not make a thorough check of the equivalence of these methods but applied both of them to the orientations that are of interest for us and ascertained that they give identical results in these cases.

The elastic compliances tensor for a cubic crystal in the standard reference frame with  axes is

For the 110-oriented plate, namely, the *x* axis along  the *y* axis along [001] and the *z* axis along [110], we obtain

where we define

For the 111-oriented plate, namely the *x* axis along , the *y* axis along  and the *z* axis along [111], we find

### A3. Bending of diamond and silicon plates

Below we use the literature values of the elastic moduli  and calculate the compliances  for the  reference frame as

We consider now two materials, diamond and silicon, which are used in spectrometers for XFELs. The elastic moduli of diamond are (McSkimin & Andreatch, 1972 ▸) , ,  and of silicon (Wortman & Evans, 1965 ▸) , ,  (all in units 10^11^ N m^−2^).

Using the compliances (32) for the 110-oriented plate, we obtain the coefficient α in equation (30) equal to  for diamond and  for silicon. The calculation for the 111-oriented plate using equation (34) gives  for diamond and  for silicon. Thus, the elastic properties of diamond give rise to an exceptionally small variation of strain over the depth *z*.

To understand the origin of the small coefficient α for the 110-oriented diamond plate, we express it through the Poisson ratio  and the Zener anisotropy ratio . Then, the coefficient α in equation (30) for the 110-oriented plate can identically be written as

In the case of an elastically isotropic crystal, one has the Lamé coefficients  and , the Poisson ratio being . Then,  and, calculating the coefficient α by equation (30), we get .

The elastic constants of diamond give  and . Both quantities are small, but not exceptionally small. However, the coefficient α is given by the difference between the Poisson and the anisotropy parameters and occurs numerically exceptionally small. For a comparison, the elastic constants of silicon give  and , so that the Poisson and the anisotropy effects only partially compensate each other.

## Appendix B Components of the scattering vector

The aim of this Appendix is to derive explicit expressions for the components of the deviation  of the scattering vector from the reciprocal-lattice vector , taking into account both an angular deviation of the incident beam  from Bragg orientation and a wavevector deviation  from the reference wavevector . We introduce the wavevectors  and , satisfying the Bragg law for the reference wavelength,  and . The wavevector of the incident wave  differs from the reference wavevector  due to both an incidence angle deviation  and a deviation of the wavevector length . Hence, the difference  is equal to

Similarly, for the wavevector of the scattered wave in the symmetric Bragg case  with its angular deviation from the reference beam direction  and the same wavevector as the incident beam , the difference  is

The components of the wavevector  =  are

It is convenient to represent the scattered intensity in an energy spectrum, considering the scattering angle  as twice the Bragg angle for the respective wavelength, *i.e.* as . Then, the components of the wavevector  are

## Appendix C Kinematical scattering amplitude for a finite width of the incident beam

Consider a Gaussian spatial distribution of the amplitude of the wave incident on the bent crystal,

where  is the distance in the direction normal to the incidence beam. This term has to be included in the integrand of equation (12), so that the amplitude of the diffracted wave for a spatially limited incidence beam can be written as

This integral can be expressed through the Faddeeva function of complex argument , where  is the complementary error function. Free codes to evaluate  are available (Poppe & Wijers, 1990 ▸; Weideman, 1994 ▸). Calculation of the integral gives

where two complex parameters which have dimensions of length

and two complex dimensionless parameters

are introduced.
